# Antimicrobial Susceptibility Patterns of Recent Cuban *Mycoplasma genitalium* Isolates Determined by a Modified Cell-Culture-Based Method

**DOI:** 10.1371/journal.pone.0162924

**Published:** 2016-09-28

**Authors:** Brian A. Mondeja, Nadia M. Rodríguez, Brenda Barroto, Orestes Blanco, Jørgen S. Jensen

**Affiliations:** 1 Pedro Kourí Tropical Medicine Institute, La Habana, Cuba; 2 Biology Faculty, University of Havana, La Habana, Cuba; 3 Microbiology and Infection Control, Statens Serum Institut, Copenhagen, Denmark; Miami University, UNITED STATES

## Abstract

Isolation of *Mycoplasma genitalium* from clinical specimens remains difficult and few strains are available for antimicrobial susceptibility testing. We describe the antimicrobial susceptibility of *M*. *genitalium* strains grown in Vero cell culture with first- and second- line antibiotics, using a modified cell-culture-based method. Macrolide- and -fluoroquinolone resistance determinants were detected by sequencing of the 23S and *parC* genes, respectively. Seven strains were examined, including three new, genetically distinct *M*. *genitalium* strains isolated from endocervical and urethral swab specimens from Cuban patients together with four reference strains isolated from specimens collected from men in Denmark, Sweden and Australia. Azithromycin was the most active drug against two of the Cuban *M*. *genitalium* strains with MICs values of 0.008 mg/liter, however, one strain was macrolide resistant with an MIC of >8 mg/liter, and the A2059G resistant genotype. Ciprofloxacin was the least active antimicrobial drug and moxifloxacin was the most active fluoroquinolone against the new clinical strains, although an MIC of 1 mg/l was found for two strains. However, no relevant *parC* mutations were detected. MICs for tetracyclines were 0.5–4 mg/liter. Although the number of Cuban strains was low, the results suggest that a single-dose azithromycin treatment could be ineffective, and that a second-line treatment with moxifloxacin, should become an option in Cuba. To our knowledge, this is the first report of isolation and antibiotic susceptibility testing of *M*. *genitalium* strains from the Latin-American region, and the first detection of macrolide resistance in such strains.

## Introduction

*Mycoplasma genitalium* is an established cause of non-gonococcocal urethritis in men and cervicitis in women [1]. Azithromycin is the most commonly used first-line treatment, but reports of treatment failure due to the rapid emergence of macrolide-resistance are now very common and widespread [2,3]. In vitro results as well as open clinical trials have shown that older fluoroquinolones as ciprofloxacin and levofloxacin are less active against *M*. *genitalium* than moxifloxacin [3]. Tetracyclines are not useful for *in vivo* eradication of *M*. *genitalium* with cure rates around 30% [4,5]. However, no genetic determinant such as *tetM* has been found in this mycoplasma species [6].

Axenic cultivation of *M*. *genitalium* directly from clinical samples is very difficult and co-culture in Vero cells appear as the most suitable alternative to obtain new strains [7]. Currently, the Vero-cell-assisted method for antimicrobial susceptibility of *M*. *genitalium* strains described by Hamasuma *et al*. in 2005 [8] is the only methodology to evaluate the susceptibility of strains not adapted to axenic growth [2,6,8,9]. Using this methodology, it is possible to obtain a susceptibility result in less than two to three months after the inoculation of *M*. *genitalium*-positive clinical samples. However, the use of Ultroser G as a serum-substitute is expensive, and the method is only available in a few laboratories in the World.

In Cuba, the recommended management of urogenital infections is syndromic. Azithromycin is the first-line drug and levofloxacin and doxycycline are the second-line treatments [10]. Since 2007, molecular diagnosis of *M*. *genitalium* infections has been performed at the “Pedro Kourí” Tropical Medicine Institute (IPK). In 2012 two new *M*. *genitalium* strains were successfully isolated and genetically characterized, showing new *M*. *genitalium mgpB* genotypes and this was the first successful isolation of this mycoplasma species in the Latin-American region [11]. However, the antimicrobial susceptibility pattern of these new strains was unknown and some concerns had been raised about the presence of macrolide-resistant strains in the Cuban population. To date, azithromycin treatment failure has been documented in 20 *M*. *genitalium* positive patients since 2014, and in June of 2015, a new strain (*mgpB* genotype 4) was isolated from one of these male patients.

The aims of the present study was to adapt and standardize the cell-assisted culture method for evaluation of the susceptibility of Cuban *M*. *genitalium* strains against macrolides, tetracyclines and fluoroquinolones. Such a method could be used in the analysis of future Cuban *M*. *genitalium* strains isolated from patients with treatment-failure and would also be an important tool in evaluation of the antimicrobial susceptibility pattern of *M*. *genitalium* strains circulating in the Latin-American Region. Preliminary data for parts of this work was presented at the 20^th^ Congress of the International Organization for Mycoplasmology, Blumenau, Brazil, 2014 (abstract No. 76).

## Materials and Methods

### *Mycoplasma genitalium* reference strains

For the cell-culture-based and broth dilution assays, *M*. *genitalium* strains isolated and characterized at Statens Serum Institut (SSI) in Denmark were used as reference strains: M2300, M2341, M6271 and M6489 [6]. Strains were inoculated into a cell culture flask with 5 mL of Vero cell suspension in 199 medium with 2% of fetal bovine serum [11]. Cell-cultures were incubated at 37°C for 21 days in 5% CO_2_. At this time, the cells were scraped off and resuspended in the supernatant. Cell suspensions were divided into 1 mL aliquots and conserved at -80°C until use. A 0.1 mL aliquot of the cell suspension was used for DNA extraction by lysis in Chelex 100 slurry [12] and a TaqMan™ PCR assay was used for *M*. *genitalium* quantitation [13].

### Cuban *M*. *genitalium* strains

Three genetically distinct *M*. *genitalium* strains isolated from Cuban patients, B3, B12 [11], and B19, were cultured in Vero cell culture following the same procedure as for the *M*. *genitalium* reference strains described above.

### *M*. *genitalium* DNA strain typing

MgPa strain typing using a 281 bp fragment of the major adhesin gene MG_191 was performed directly from the clinical samples giving rise to the Cuban strains B3, B12, and B19 and from the inoculum used for the antimicrobial susceptibility testing as previously described [14]. The *M*. *genitalium* reference strains isolated in Denmark had undergone the same procedure previously and the typing was not repeated.

### Antimicrobial susceptibility assays

#### Antimicrobials

The antimicrobials for susceptibility testing were azithromycin (Novatec, Cuba), erythromycin (Sigma, USA), ciprofloxacin (Sigma, USA), ofloxacin (Sigma, USA), levofloxacin (Novatec, Cuba), moxifloxacin (Bayer, Italy), tetracycline (Sigma, USA) and doxycycline (Bayer, Italy). All compounds were diluted and conserved according to the specifications in the CLSI-Guideline M43-A: “Methods for Antimicrobial Susceptibility Testing for Human Mycoplasmas” [15].

#### Cell-culture-based antimicrobial susceptibility test

The antimicrobial susceptibility assays were performed using a modification of the Hamasuma *et al*. protocol [8]. Briefly, Vero cells grown in 50-ml flasks were trypsinized and resuspended in 199 medium with 2% foetal bovine serum and adjusted to 1.7 x 10^4^ cells/ml. After thawing, *M*. *genitalium* strains propagated in Vero cell culture were diluted in cell culture medium and adjusted to contain from 3 x 10^3^ to 1 x 10^4^ genome equivalents (geq) of *M*. *genitalium* per 0.1 ml, and 0.1 ml of the diluted inoculum was dispensed into the wells of Multiwell 96-well tissue culture plates (Becton Dickinson, France). Each antibiotic was diluted with 199 medium without foetal bovine serum in twofold steps, and 0.1 ml of the dilution was added to each well. Triplicate control wells received 0.1 ml of the same medium without antibiotics resulting in a total volume of 0.2 ml medium in each well. The plates were covered with sterile sealing tape (Nunc, Roskilde, Denmark) to prevent evaporation of the medium and incubated in an atmosphere with 5% CO_2_ at 37°C. At 2 weeks after the inoculation, 0.1 mL of the supernatant was harvested from each well and added to 0.3 mL of 5% Chelex 100 slurry, and the *M*. *genitalium* DNA load was determined by the *M*. *genitalium mgpB* TaqMan PCR assay [12]. Inhibition rates of the antibiotics were calculated by the formula: inhibition rate (%) = [(average of DNA loads in control wells -DNA load in test well)/(average of DNA loads in control wells)]x 100. The MIC was defined as the lowest concentration of antibiotic causing 99% inhibition.

### Detection of molecular markers of macrolide and quinolone resistance

Molecular markers of macrolide resistance were detected by sequencing of a fragment of region V of the 23S rRNA gene from genomic DNA of an early passage of the strains [2]. Markers of fluoroquinolone resistance were detected by sequencing a fragment of the *parC* gene containing the quinolone resistance-determining region (QRDR) [16].

## Results

### *M*. *genitalium* DNA strain typing

Each of the newly isolated Cuban *M*. *genitalium* strains had a different MgPa sequence type. Identical sequence types were found in the original clinical sample and in the isolated strain used for inoculum in the antimicrobial susceptibility test, thus documenting absence of cross-contamination.

### Cell-culture-based antimicrobial susceptibility test

MIC values of the *M*. *genitalium* reference strains determined in the modified cell-culture assay were similar to the values reported in the literature for these strains in the original cell-culture system [8,9]. Table 1 shows the MIC values for the strains tested. Azithromycin was the most active drug against two Cuban *M*. *genitalium* strains with MICs values of 0.008 mg/l and one strain was detected as macrolide resistant with a MIC of >8 mg/l. Ciprofloxacin was considerably less active than moxifloxacin, which remained the most active fluoroquinolone against the new clinical strains. MICs for tetracyclines were 0.5–4 mg/l and as expected, doxycycline had a lower MIC than tetracycline.

**Table 1:** Minimal Inhibitory Concentration values for *M*. *genitalium* strains determined by a modified cell-culture-based method.

*M*. *genitalium* strain	MIC VALUES (mg/L)							
	Azithromycin	Erythromycin	Ciprofloxacin	Ofloxacin	Levofloxacin	Moxifloxacin	Tetracycline	Doxycycline
M6271	>8	>8	1	1	1	0.125	0.25	0.5
M2300	<0.008	<0.008	2	1	4	0.25	0.25	1
M2341	<0.008	<0.008	1	0.5	0.25	0.125	0.25	0.25
M6489	>8	>8	>8	8	>8	>8	2	2
B3	<0.008	<0.008	4	4	0.5	0.25	2	2
B12	<0.008	<0.008	4	1	1	1	4	0.5
B19	>8	>8	2	1	1	1	4	0.5

### Detection of molecular markers of macrolide and quinolone resistance

The 23S rRNA gene sequences were in accordance with the MIC results for macrolides. No mutations were detected in region V of the 23S rRNA gene in the two macrolide susceptible strains (MIC 0.008 μg/ml; B3 and B12), but an A2059G (*Escherichia coli* numbering) transition was detected in the phenotypically macrolide resistant B19 strain. No mutations were detected in the QRDR of the *parC* gene in the Cuban *M*. *genitalium* isolates.

## Discussion

We modified the cell culture-based antimicrobial susceptibility assay [8] in terms of the growth medium which was changed to a low percentage of fetal bovine serum instead of the Ultroser G serum-free supplement which is expensive and difficult to source in many settings. The 24-well plate format was also exchanged for the standard 96-well microtiter plate format. Both modifications led to significant cost-savings and a higher throughput. Based on comparison with the MIC values obtained with the original method for four well-characterized *M*. *genitalium* strains, we also decided to shorten the incubation period from three to two weeks. This modification also led to increased throughput. The use of the microtiter plate cell-culture system based on Vero cells with 199 medium and fetal bovine serum revealed comparable MICs with the Vero-system using Ultroser G.

Currently, no approved guideline for antimicrobial susceptibility testing exists for fastidious mycoplasmas of human origin such as *M*. *genitalium*, *M*. *fermentans* and *M*. *penetrans*, whereas such guidelines have been developed for human species capable of axenic growth such as *M*. *pneumoniae*, *M*. *hominis* and the ureaplasmas [17]. However, with the increasing importance of *M*. *genitalium* as a multi-drug resistant sexually transmitted infection [3], it is important to start defining media formulations and/or procedures for a future establishment of such guidelines. This will involve collaborative inter-laboratory studies with large numbers of strains, before such a consensus can be reached. Consequently, an improvement of the isolation procedure is needed because the currently used co-culture in Vero cell is time-consuming and complex to perform.

Two of the available Cuban *M*. *genitalium* strains were susceptible to azithromycin and this correlates well with the clinical history of the corresponding patients, as azithromycin in the five-days regimen was effective in both cases. The number of Cuban strains analyzed in the present study, however, is too low to establish an estimate of the prevalence of macrolide resistance in the population. The B19 strain showed a MIC value >8 mg/L for azithromycin and an A2059G mutation explained the resistance. The corresponding patient failed several courses of azithromycin treatment over seven months, again showing a strong correlation between macrolide resistance and clinical treatment failure. He was eventually cured by a combination of levofloxacin and tetracycline.

Clinical experience also suggest that macrolide resistance may be common in Cuba based on several cases of treatment failure after azithromycin 1 g syndromic treatment. In recent years, emergence of resistance to macrolides has been recorded around the world [18–22], but this is the first report from the Latin-American region. The A2059G transition is the second-most common mutation in Europe, Asia and Australia [18–22] and some studies have associated this mutation with higher MICs values for some macrolide drugs [6].

Interestingly, the MICs for tetracyclines in the Cuban strains were rather high. This correlates well with the clinical information at least from the woman that provided the sample containing the B3 strain. This patient had been treated for chronic cervicitis and infertility with an extended doxycycline regimen without clinical improvement 21 days before the sampling for *M*. *genitalium*.

The older fluoroquinolones evaluated in the present study had an elevated MICs value of 1–4 mg/liter suggesting decreased susceptibility or resistance. The MIC values for moxifloxacin was also surprisingly high. Only the B3 strain showed a MIC of 0.25 mg/L which could be classified as susceptible. The other two strains had MICs of 1 mg/L, suggesting a decreased susceptibility to this drug although accepted breakpoints have not been established. However, the apparent elevated quinolone MICs were not explained by sequencing analysis of the QRDR of the *parC* gene as mutations known to confer quinolone-resistance were not detected [22–24]. The class *Mollicutes*, including *M*. *genitalium*, is phylogenetically closely related to the low-GC Gram-positive bacteria, thus, the primary targets for quinolone resistance mutations are in the *parC* gene. We did not sequence the *gyrA* gene of the strains, as mutations in this gene have never been associated with moxifloxacin treatment failure. However, this will be performed along with control experiments controlling the influence of incubation for two versus three weeks for these less well adapted *M*. *genitalium* strains.

Based on our findings and the experience from other settings, we suggest the need to revise the Cuban treatment guidelines with the elimination of the single-dose azithromycin treatment, which has been shown to be a strong selection mechanism for resistance. For patients with macrolide-susceptible strains, the extended five day azithromycin regimen should be used [3] and macrolide resistant strains or patients experiencing treatment failure could be treated with an extended regimen of doxycycline plus levofloxacin as alternative treatment until the availability of moxifloxacin in Cuba is ensured.

The good concordance between the results of this modified assay and the previously published method found for the reference strains suggest that the modified assay can be used to decrease the cost by using a common medium and serum in low quantities and reduced volumes for determination of MICs.
